# Evaluation of a novel educational intervention for mental health staff on advance care planning with older people with mental illness

**DOI:** 10.1007/s41999-026-01416-y

**Published:** 2026-01-27

**Authors:** Keanu Crous, Daniella Kanareck, Margaret Anne Thomas, Kate Anderson, Simon Tully, Anne Pamela Frances Wand

**Affiliations:** 1 Faculty of Medicine and Health, University of Sydney, c/o Camperdown Older Peoples Mental Health Service, Missenden Rd, King George V, Level 7, Camperdown, NSW 2050 Australia; 2 Eastern Suburbs Older Persons Mental Health Service, New South Wales, Australia; 3 Mental Health Service, Sydney Local Health District, New South Wales, Australia; 4 Specialty of Psychiatry, Faculty of Medicine and Health, University of Sydney, Sydney, Australia; 5 School of Psychiatry and Mental Health, Faculty of Medicine and Health, University of New South Wales, Sydney, Australia

**Keywords:** Education, Advance directives, End-of-life, Mental disorder, Mental health, Psychogeriatrics

## Abstract

**Purpose:**

To evaluate a novel educational intervention for mental health clinicians and peer workers on Advance Care Planning (ACP). The aims of the intervention were to improve staff knowledge, attitudes, and confidence in facilitating ACP with older people with mental illness and increase staff engagement in ACP.

**Methods:**

A pre–post-intervention study was conducted across two public mental health services in Australia. Multidisciplinary clinicians and peer workers (*n* = 110) participated in a one-hour, evidence-informed educational session combining theory, video demonstrations, and discussion of case vignettes. Uniquely, this study utilized purpose-developed audio–visual resources designed for local legal frameworks and engagement of peer workers as both educators and participants. Paired pre- and post-intervention questionnaires assessed staff knowledge, attitudes, and confidence. A pre–post-audit of mental health consumer (patient) electronic medical records (eMR) quantified changes in ACP documentation rates. Thematic analysis of free-text feedback was conducted.

**Results:**

The intervention significantly improved staff attitudes (*p* < 0.001, *r* = 0.48), knowledge (*p* < 0.001, *r* = 0.51), and confidence (*p* < 0.001, *r* = 0.87). However, eMR audits (pre: *n* = 1104; post: *n* = 1118) with a 2–3-month follow-up period revealed no significant change in ACP documentation rates (7.6% vs. 8.0%). Participants’ feedback highlighted a desire for further practical training incorporating experiential learning (e.g., role-play) and systemic support.

**Conclusions:**

While a brief, standalone educational intervention significantly improved staff readiness for ACP, this did not translate into documented changes in clinical practice (eMR documentation). Bridging this knowledge-to-practice gap requires multifaceted strategies beyond standalone education, such as organizational governance, audit-feedback cycles, skills practice, and champions to embed ACP into routine practice.

**Supplementary Information:**

The online version contains supplementary material available at 10.1007/s41999-026-01416-y.

## Key summary points

**Aim:**

To evaluate whether a brief educational intervention for mental health staff on Advance Care Planning (ACP) improves attitudes, knowledge, confidence, and engagement in ACP with older people with mental illness.

**Findings:**

The intervention significantly improved staff readiness for ACP and was well received, but did not significantly increase ACP documentation rates in the brief follow-up period.

**Message:**

Additional and ongoing strategies, such as organizational change, skills practice, audit, and feedback, may be required to effect staff behavioral change with ACP engagement and documentation.

**Supplementary Information:**

The online version contains supplementary material available at 10.1007/s41999-026-01416-y.

## Introduction

Advance care planning (ACP) supports adults in understanding and sharing their values and preferences for future medical care [1]. Benefits of effective ACP include extending patient autonomy, improved patient and family satisfaction, reduced emotional burden, increased use of hospice and palliative care, and a higher likelihood of receiving goal-concordant care [2, 3]. This process is particularly important for people with serious mental illness as ACP enables them to state their treatment preferences while capacitous. Yet despite these recognized benefits, access to ACP is suboptimal, particularly for people living with mental illness. This population experiences reduced life expectancy, largely due to high rates of chronic co-morbid physical illnesses [4, 5], and poorer end-of-life outcomes, including undertreated physical symptoms [5, 6]. Adverse outcomes may be compounded for older people with mental illness, given the documented disparities in access to healthcare and social support [5]. Thus, while people with mental illness may have greater need for ACP due to their complex health profiles and shortened life expectancy, they paradoxically have less access to these crucial conversations.

Recent efforts to address the gap in ACP for people with mental illness (referred to here as consumers) have included exploring perspectives of consumers and their carers [3, 7] and mental health clinicians [8, 9], discussion of complex cases [10], and developing targeted resources [11]. These studies highlight that consumers or their carers may lack awareness regarding ACP, reporting clinicians never raised it with them [3]. Carers [3] and clinicians [9] may be reluctant to engage in ACP with older people living with serious mental illness due to perceived complexity and fear of causing distress. Clinicians report additional barriers including lack of knowledge and training, systemic issues, such as the fragmentation of care between services [9, 12], a lack of policies to guide practice, insufficient time for ACP conversations [9], and uncertainty about decision-making capacity and choosing the timing of ACP discussions [9]. Clinicians may also be influenced by mentalism (discrimination on the basis of mental illness) in their appraisal of outcomes and aspects of care toward the end-of-life [13]. To date, these rich data have not been used to inform and test approaches to implementation of ACP in mental health settings.

Educational interventions represent a primary strategy to address these clinician-level barriers, but no such interventions have been described targeting mental health clinician and peer worker engagement in ACP with older people living with mental illness. While a US-based, multifaceted demonstration project focusing on mental health patients described positive outcomes over 20 years ago [14], there are no subsequent reports regarding its sustainability and the details of the intervention itself are unavailable, precluding replication. Considering the paucity of research in the mental health context, the broader yet somewhat limited literature regarding ACP with older people was examined, revealing a fragmented evidence base. While some interventions successfully increased documentation albeit without assessing underlying clinician learning [15], others improved staff self-efficacy but failed to translate into improved knowledge or practice change [16]. The latter exemplifies the ‘knowledge-to-practice’ gap, a well-documented phenomenon in healthcare professional education where validated increases in clinician knowledge, confidence or self-efficacy may fail to translate into observable changes in clinical behavior [17, 18], emphasizing the need to evaluate both learning and implementation outcomes simultaneously.

### Objective

Building on prior qualitative research exploring clinician, consumer, and carer perspectives on ACP for older people living with serious mental illness in Australia [3, 9, 13], this study evaluated a novel evidence-informed educational intervention on ACP for mental health clinicians and peer workers. Specifically, the aims were to (i) improve clinician and peer worker knowledge, attitudes, and confidence in facilitating ACP discussions with older people living with serious mental illness and (ii) to improve staff engagement in ACP, measured indirectly by electronic medical record (eMR) documentation.

## Methods

### Study design

This pre–post-intervention study evaluated an evidence-based educational intervention on ACP for mental health clinicians and peer workers working with older people with mental illness. Second, this study employed mixed methods, combining quantitative assessment of knowledge, attitudes, and confidence via questionnaires with objective evaluation of clinical practice change through eMR audits. Third, this study complied with STROBE guidelines (https://www.strobe-statement.org/). Fourth, this study was approved by the Concord Hospital Human Research Ethics Committee (2023/ETH02283).

### Setting

This study was conducted across two public Mental Health Services in two government-funded Health Districts in Sydney, Australia: Sydney Local Health District (SLHD) Mental Health Service, and the Eastern Suburbs Older Persons Mental Health Service, South Eastern Sydney Local Health District (SESLHD). SLHD is located in central Sydney, covering a geographical area of 126 km with a population of approximately 740,000 people (https://slhd.health.nsw.gov.au/organisation). SESLHD covers an area of 468 km, serving a population of over 930,000 residents in the Eastern suburbs of Sydney (https://www.seslhd.health.nsw.gov.au/services-clinics/directory/about-us). Both Mental Health Services provide individualized care coordination through multidisciplinary teams comprising mental health clinicians from diverse professional backgrounds and peer workers, delivering care across both hospital and community settings (https://slhd.health.nsw.gov.au/mental-health/community; https://www.seslhd.health.nsw.gov.au/services-clinics/mental-health).

### Participants

Staff participants: All multidisciplinary mental health clinicians and peer workers employed at the study sites were eligible for inclusion. Staff were recruited through existing multidisciplinary and peer worker in-service/education programs, in a combination of in person only, hybrid, and online only formats. All sessions were facilitated by the lead investigator (AW) and another member of the research team, matched to the learner audience (e.g., nursing co-facilitator for nursing in-services). Clinicians and peer workers were invited to participate in evaluation of the intervention (i.e., questionnaires) at the start of each education session.

Consumer eMR audit: The eMR audit included all active (non-discharged) consumers aged 55 years and older receiving mental health services from the study sites. These consumers were under the care of staff targeted by the intervention and the focus of ACP engagement.

### Educational intervention

The content of the educational intervention was directly informed by the research team’s previous studies exploring the barriers and facilitators to ACP with people with mental illness [3, 9] and relevant literature [12, 14, 19]. The one-hour structured session consisted of theoretical content, purpose-developed video excerpts demonstrating aspects of conducting ACP conversations with older mental health consumers and their carers, and interactive discussion of case vignettes (see Supplementary Material). The educational content and resources were designed specifically for the Australian context, incorporating principles of guardianship and supported decision-making frameworks, which vary by state and territory in Australian jurisdictions [20]. In New South Wales, where this study was conducted, the legal appointment of a substitute decision-maker for health and lifestyle decisions is termed an 'Enduring Guardian'. The Enduring Guardian role is functionally equivalent to a Durable Power of Attorney for Healthcare (e.g., some parts of the USA) or Lasting Power of Attorney for Health and Welfare (Wales, England) or Healthcare Proxy (e.g., Austria, Germany, and some parts of the USA). Decision-making models, including substitute decision-making and supported decision-making, vary internationally and across jurisdictions; Australia has increasingly emphasized supported decision-making approaches that prioritize an individual’s will and preferences [20].

Tailored one-page summary handouts on ACP for each group (clinicians, carers, consumers) and links to these (including Arabic, Greek and Chinese translations) and other online resources for ACP, such as the full training videos, were provided. These resources were previously developed by the research team [11], informed by the original empirical qualitative studies [3, 9, 13].

### Questionnaires

As there was no suitable published validated questionnaire to assess learning in ACP, one was developed to assess clinician/peer worker knowledge, attitudes and confidence regarding ACP with mental health consumers. The pre- and post-intervention versions of the questionnaires included the same domains of attitude (5 items), knowledge (8 items), and confidence (5 items). The pre-intervention questionnaire included demographic questions (age, gender, discipline, work setting, prior relevant experiences). The post-intervention questionnaire included two questions on intention to engage in ACP, and two free-text questions seeking feedback on the intervention; “What did you find most useful from the education session?” and “How could the education session be improved?’. The questionnaire was presented to a convenience sample of clinicians and a peer worker for feedback, with changes incorporated to ensure face validity.

Questionnaires were completed anonymously by participants immediately before and after the educational intervention and individual responses paired through a unique code.

### Electronic medical record (eMR) documentation

Audits of ACP documentation on eMR were conducted before the educational intervention sessions and repeated 2–3 months later. Data gathered at the two time points included the presence of an ACP or an Advance Care Directive (ACD)^1^ document, an Enduring Guardian document, documentation of ACP discussions, an active resuscitation document and any ACP on HealtheNet (a State-wide clinical portal), alongside aged care facility residency status, and inpatient status.

### Statistical analysis

Two-sided p < 0.05 was defined as statistically significant for all analyses. 95% confidence intervals were calculated for mean differences using the t distribution and for proportions using normal approximation.

(i) Questionnaire data: Participant demographics were summarized using means (SD) for continuous variables and frequencies (%) for categorical variables. Due to non-normal distributions and ceiling effects identified in preliminary data inspection, non-parametric tests were employed: Domain scores were compared using Wilcoxon signed-rank tests with effect sizes calculated using Rosenthal’s r (small: 0.1–0.3, medium: 0.3–0.5, large: > 0.5). Individual items used Exact McNemar tests with a Bonferroni correction for multiple comparisons (*n* = 18). Exploratory analysis used Mann–Whitney *U* tests to examine whether outcomes differed by delivery mode, age (< 35 or 35 +), or prior experience.
(ii) eMR Documentation: The proportion of mental health consumers aged 55 + with any ACP-related documentation was calculated at both timepoints, pre- and post-educational intervention, to examine change in staff practice of engaging in and documenting ACP. Change in documentation rates was tested using two-sample z test for proportions.
(iii) Qualitative Analysis: Responses to the free-text feedback questions were thematically analyzed following Braun and Clarke’s method [21]. This flexible process identifies, analyzes, and reports patterns, or 'themes', within qualitative data that capture important aspects related to the research question. Two independent coders (AW, KC) performed this analysis.

## Results

### Participant characteristics and response rates

Six education sessions on ACP were conducted across the study sites between April and May 2025, reaching 110 mental health staff (50 online, 60 in-person). The overall response rate for questionnaire completion was 79% (87/110) and differed between delivery modes: 100% for in-person participants versus 54% for online participants.

Of the 87 respondents, 73 (84%) completed both the pre- and post-intervention questionnaires, enabling paired analysis. After excluding questionnaires with incomplete domain data, the final sample ranged from 66 to 69 paired questionnaires depending on the specific domain analyzed. Demographics for the total sample and the analyzed sample are presented in Table 1.

**Table 1:** Demographics of staff participants

Characteristic	Total sample (*n* = 87)		Analyzed sample^a^ (*n* = 73)	
Age (years)	*n* = 78		*n* = 65	
Mean (SD)	39.2	(12.8)	38.8	(12.6)
Gender, *n* (%)	*n* = 86		*n* = 73	
Female	64	(74.4%)	55	(75.3%)
Male	21	(24.4%)	17	(23.3%)
Other	1	(1.2%)	1	(1.4%)
Discipline, *n* (%)	*n* = 86		*n* = 73	
Medical	29	(33.7%)	27	(37.0%)
Nursing	25	(29.1%)	22	(30.1%)
Social Work	14	(16.3%)	8	(11.0%)
Peer Support	9	(10.5%)	7	(9.6%)
Psychology	7	(8.1%)	7	(9.6%)
Occupational Therapy	2	(2.3%)	2	(2.7%)
Experience, *n* (%^b^)	*n* = 83		*n* = 70	
Older adult (55 +) Mental Health	76	(91.6%)	65	(92.9%)
Prior ACP Training	9	(10.8%)	7	(10.0%)
Prior ACP Experience	23	(27.7%)	18	(25.7%)

^a^Participants who completed both pre- and post-education questionnaires and were included in at least one analysis

^b^Percentages add to over 100% as participants could choose multiple answers for aspects of prior ACP Experience

No significant differences were found when baseline domain scores were compared between participants who completed only the pre-intervention questionnaire (*n* = 13) versus those with complete paired data (*n* = 73) across any domain (all *p* > 0.315).

### Changes in domain scores (knowledge, attitudes, and confidence)

The educational intervention resulted in statistically significant improvements across all three domains (Table 2). The largest effect size observed was for confidence (*p* < 0.001, *r* = 0.87), indicating substantial improvement in participants’ self-rated ability to engage in ACP discussions. Knowledge also showed a large effect (*p* < 0.001, *r* = 0.51), while attitude improvements showed a medium effect (*p* < 0.001, *r* = 0.48).

**Table 2:** Pre- and post-intervention scores by domain

Domain (correct response given)	Attitude (5 items) (*n* = 68)	Knowledge (8 items) (*n* = 66)	Confidence (5 items) (*n* = 69)
Pre-mean (SD)	4.25 (0.82)	6.71 (1.22)	1.23 (1.32)
Post-mean (SD)	4.77 (0.46)	7.35 (0.94)	4.32 (1.05)
Mean Δ (SD)	0.52 (0.91)	0.64 (1.1)	3.09 (1.32)
% Pre-mean (SD)	85.0% (16.3%)	83.9% (15.3%)	24.6% (26.4%)
% Post-mean (SD)	95.3% (9.2%)	91.9% (11.7%)	86.4% (21.0%)
% Mean Δ (SD)	10.3% (18.1%)	8.0% (13.8%)	61.7% (26.3%)
Mean Δ 95% CI	0.30, 0.73	0.23, 0.57	2.77, 3.40
	5.9%, 14.7%	4.6%, 11.3%	55.4%, 68.1%
Test statistics (Z)	3.989	4.131	7.25
*p* value*	< 0.001	< 0.001	< 0.001
Effect size (*r*)	0.484 (med)	0.509 (large)	0.873 (large)

^*^p value calculated using WSR test (2-tailed)

### Item analysis

An analysis of individual questions (Table 3) showed large but non-uniform improvements across most items. Significant increases were seen in two knowledge items, all confidence-related items and one attitude item (Table 3). For the two intention items, there were 68 respondents, with 60 intending to both engage in ACP discussions with mental health consumers and use the handouts, three to engage in ACP but not use the handouts, and five to use handouts but not engage in ACP (Table 3).

**Table 3:** Questionnaire items, pre–post-intervention

Question and Answer [Agree / Disagree OR Multiple Choice]—Correct Answer is Bolded				Correct (n)		*p*-value *	Adj. *p*-value **	
				Pre	Post			*N*
Attitude	1	ACP is only relevant for people with serious medical illnesses [**Disagree**]		63	70	0.092	N/S	73
	2	Discussing ACP with mental health consumers is part of my role [**Agree**]		56	70	< 0.001	0.009	71
	3	Discussing ACP with mental health consumers damages the therapeutic relationship [**Disagree**]		69	72	N/S	N/S	73
	4	It takes too long to do ACP with consumers [**Disagree**]		55	61	N/S	N/S	70
	5	A person with schizophrenia can make decisions about their end-of-life care [**Agree**]		65	70	N/S	N/S	72
Knowledge	6	Next-of-kin can override a consumer's Advance Care Directive (ACD) [**Disagree**]		56	69	< 0.001	0.018	73
	7	If a consumer is cognitively impaired, they cannot make an ACP [**Disagree**]		43	60	< 0.001	0.004	70
	8	Nominated family members should be involved in ACP discussions with a consumer [**Agree**]		68	71	N/S	N/S	73
	9	Once written, an ACP cannot be changed [**Disagree**]		71	72	N/S	N/S	73
	10	If a consumer lacks capacity, supported decision-making can be used to facilitate ACP [**Agree**]		68	72	N/S	N/S	73
	11	A 57-year-old woman with bipolar disorder is involuntarily admitted with a relapse of mania characterized by delusions that she is a medical professor who can cure illness through targeted eye-blinking, delusions of reference when reading books, and disorganized thoughts. During an eMR audit it is noted that she does not have any ACP documentation. Which one of the following is most correct?	**a. Defer the ACP discussion until her mania has improved** b. As she is an involuntary patient it is not appropriate to discuss ACP c. Given her age and absence of physical comorbidities an ACP is not needed d. Discuss and document ACP with the patient now	54	61	N/S	N/S	70
	12	A 76-year-old man with dementia has been receiving treatment from the community mental health team for depressive symptoms over the last 8 months. He has prostate cancer and is receiving hormone therapy. His wife wants him to make an ACP. Which one of the following is most correct response?	a. Let her know this is not possible as he has lost capacity b. Ask her to write an ACP on his behalf c. Recommend waiting until his depressive symptoms have fully resolved **d. Engage the man in ACP using supported decision-making**	59	56	N/S	N/S	71
	13	A 61-year-old man with longstanding schizophrenia is receiving care and a depot antipsychotic under a community treatment order due to poor adherence to medication and lack of insight. He has chronic psychotic symptoms despite treatment. He has a sedentary lifestyle and smokes but is not known to have any physical comorbidities. He rarely sees his general practitioner. Which one of the following actions is most appropriate for his mental health case manager in relation to ACP?	a. No action is required as he does not need an ACP **b. Discuss the optimum timing of ACP with the multidisciplinary team** c. Request his GP write an ACP d. No action. He cannot be engaged in ACP due to chronic psychosis	63	67	N/S	N/S	69
Confidence	14	I feel confident initiating ACP discussions with consumers		19	54	< 0.001	< 0.001	69
	15	I feel confident involving families/carers in ACP discussions		30	58	< 0.001	< 0.001	71
	16	I know where to document ACP discussions on the eMR		19	70	< 0.001	< 0.001	72
	17	I know how to determine the optimum timing for ACP		8	56	< 0.001	< 0.001	71
	18	I know where to find information on ACP with people with mental illness		9	68	< 0.001	< 0.001	71

*ACP* Advance Care Plan / Planning, *ACD* Advance Care Directive, *eMR* Electronic Medical Record, *GP* General Practitioner, *N/S* non-significant, *N* number of eligible respondents

^*^P-value calculated using exact McNemar’s test (2-tail)

^**^Bonferroni adjusted to reflect 18 tests

### Exploratory subgroup analysis

There were no statistically significant differences in score improvement between age groups or modes of participation. Participants with no prior experience or training showed significantly greater improvements in both knowledge (*p* = 0.042) and confidence (*p* = 0.017) compared to those with prior experience.

### Thematic analysis of qualitative feedback

Emergent themes from thematic analysis of free-text responses (*n* = 60 for “most useful”; *n* = 33 for “improvements”) are presented in Table 4 with illustrative quotations. Overall, the feedback on the educational session was very positive, with remarks, such as “*very beneficial*” (female, nursing), “*very informative, got me thinking*” (male, peer support worker), and “*great first introduction for the available time*” (female, psychology). Themes for the most useful aspects of the education were ‘Presentation delivery’; ‘Practical application’; ‘Theoretical content’; and ‘Empowering’. Themes for how the education could be improved were ‘Presentation delivery’; ‘Broader demonstration of clinical practice’; and ‘More resources’.

**Table 4:** Thematic analysis of education session feedback

Core theme	Illustrative quote
*Most useful aspects of the education (n = 60)*	
(i) Presentation delivery	"Very clear, engaging presenter. Useful handouts. Interesting vignettes." (male, medical)
	"The content is important and was easy to understand" (female, nursing)
	“Great to have the discussion” (female, psychology)
(ii) Practical Application	"The handouts, vignettes and videos were handy to demonstrate process and answer common barriers to completing ACPs" (nonbinary, medical)
	“Where to document ACP on EMR and ACD.” (male, nursing)
(iii) Theoretical content	"Differences between ACP and ACD. Capacity assessment in patients with mental illnesses." (female, medical)
	“Identification of key issues/concerns” (male, nursing)
(iv) Empowering	“It was helpful to know that this [ACP] is an option to give people a voice in their care.” (female, peer worker)
*How the education could be improved (n = 33)*	
(i) Presentation delivery	One participant noted the session was too long, while others requested more time. Technical issues like poor sound quality and the presentation starting early were also mentioned
(ii) Broader demonstration of clinical practice	"More examples of challenging situations with patients with chronically poor therapeutic relationships." (male, medical)
	"More examples of phrases to use to start the discussion; how to respond when psychotic symptoms are raised by patient during ACP" (female, psychology)
	"Further elaboration on capacity assessment. Discussion on resuscitation plan for inpatient and what to do in emergency scenarios." (male, nursing)
	“Show an example of an ACP/what is documented in EMR” (female, medical)
(iii) More resources	"Standardized handouts to give to patients > laminated cards to put on lanyards." (female, medical)
	"Extra session on what to discuss in advanced care planning to help train and build confidence in all clinicians." (female, medical)

*ACP* Advance Care Plan / Planning, *ACD* Advance Care Directive, *EMR* Electronic Medical Record

### Change in clinical practice: eMR documentation rates

The eMRs of all eligible consumers at both study sites were audited for ACP documentation (Table 5). Pre-intervention 84/1104 (7.6%) consumers had some form of ACP-related documentation in their eMR compared to 89/1118 (8.0%) post-intervention. A two-proportion z test showed no significant difference (*p* = 0.6643).

**Table 5:** ACP-related documentation in mental health consumer electronic medical records

Document type	Pre-intervention *N* = 1104 | n (%)	Post-intervention *N* = 1118 | n (%)	p value^*^	Delta %	95% CI
ACP / ACD	28 (2.5%)	37 (3.3%)	0.051	0.77%	−0.7%, 2.2%
Enduring Guardian	18 (1.6%)	13 (1.2%)	0.890	-0.47%	−1.5%, 0.5%
Discussion Notes	47 (4.3%)	49 (4.4%)	0.419	0.13%	−1.6%, 1.8%
HealtheNet	2 (0.2%)	3 (0.3%)	0.247	0.09%	−0.3%, 0.5%
Resuscitation Plan^a^	7 (0.6%)	6 (0.5%)	0.659	-0.10%	−0.7%, 0.5%
Any ACP-specific documentation^b^	66 (6.0%)	73 (6.5%)	0.222	0.55%	−1.5%, 2.6%
Any ACP-related documentation^c^	84 (7.6%)	89 (8.0%)	0.332	0.35%	−1.9%, 2.6%
**Setting factors**					
Living in a nursing home	98	110			
Current mental health inpatient	68	95			

^a^Resuscitation plan is only documented for inpatients

^b^Any of ACP, ACD or ACP Discussion Notes

^c^Any of the above

^*^p values calculated using Z-test (1-tail)

*ACP* Advance Care Plan / Planning, *ACD* Advance Care Directive, *eMR* Electronic Medical Record

N.B. The sample sizes (**N**) are unequal as they reflect the number of active (non-discharged) consumers at each timepoint

## Discussion

To our knowledge, this is the first evaluation of a dedicated educational intervention for mental health clinicians and peer workers on ACP with people with mental illness. This intervention was novel in its specific tailoring to the Australian medicolegal context and its integration of the lived experience workforce (peer workers) as both educators and learners. Following this brief intervention, there were significant improvements in mental health staff attitudes, knowledge and confidence regarding ACP with older people living with serious mental illness. Confidence items showed the most dramatic improvements, and qualitative feedback indicated that staff felt empowered to view ACP as part of their role, addressing previous reservations [9]. However, despite gains in readiness to engage in ACP, the prevalence of ACP-related documentation in mental health consumer eMRs following the intervention remained stable and low (7.6% to 8.0%).

The US-based “Do It Your Way” project provides the most relevant comparison of an educational initiative to improve ACP with people with mental illness [14]. They too demonstrated improvements in provider knowledge and confidence, but, in contrast to our study, they achieved increased health care proxy rates post-intervention from a baseline of 0.3%, reaching 1% at the one-year follow-up, 2.2% at two years, and 4.7% at three years. Their success may be explained by their multifaceted approach involving a coalition of stakeholders, community and patient outreach, and mandated performance metrics, rather than staff education alone [14]. Furthermore, they measured outcomes over three years, whereas our study was limited to a 2–3-month follow-up. This suggests that while standalone education is a necessary step, it is insufficient to drive practice change in isolation. Similarly, a study focused on mental health consumers found that education improved their knowledge and attitudes regarding ACP but not the intention to sign an ACD [22].

Our results mirror the broader ACP literature in non-mental health settings. For example, a systematic review of training programs for healthcare professionals found that while education consistently improves knowledge, attitudes, and perceived communication skills, there is insufficient evidence that it increases the frequency of ACP discussions, often attributed to workload and insufficient time [23]. Crucially, the literature suggests that the design of multi-component interventions determines their success. For example, a US-based primary care study focusing on older adults achieved a significant increase (1.4 times baseline) in ACP documentation by combining interprofessional education (including role-play) with workflow redesigns and ongoing monthly coaching over 12 months [15]. Conversely, a train-the-trainer model in nursing homes which incorporated coaching, conversation guides, audit and multidisciplinary team meetings did not improve staff knowledge or enhance documentation despite improved staff self-efficacy [16]. The negative findings were attributed to implementation challenges and short follow-up [16]. These contrasting outcomes suggest that while education alone is insufficient, multi-component interventions may require robust implementation support, such as workflow integration and coaching, to drive practice change. These findings from the general ACP literature provide valuable lessons for translation into mental health settings, with some strategies also suggested by clinicians in our previous qualitative research [9].

The discrepancy between increased staff readiness but stagnant documentation rates, the “knowledge-to-practice” gap, may be explained by several factors. It is possible that consumers who were at the younger end of the 55 + age range, who considered themselves physically well lacked impetus to engage in ACP [24], or their clinicians considered ACP not indicated for these reasons, although these explanations did not emerge in our initial qualitative studies [3, 9]. Alternatively, ACP discussions may have occurred but not been documented, noting clinician reports of time pressure [9], but perhaps also due to uncertainty about how and where to document these discussions (see Table 4). Although evidence-informed educational resources were created [11] and freely available to participants to support implementation, their uptake/access over the follow-up period is unknown. The knowledge-to-practice gap may also be due to systemic factors [9, 12] rather than the educational intervention itself. In contrast to multi-component strategies described above [14, 15, 25], aside from Mental Health Executive support, the present study was not accompanied by systemic change in the mental health service, such as through guidelines, policy and ongoing audit, and feedback. These strategies have been described as essential in other complex areas of healthcare such as delirium [26, 27]. Qualitative feedback revealed that while staff grasped the theory, they were less confident with the mechanics of ACP conversations. Participants requested role-plays and “broader demonstrations of clinical practice”, reflecting the limitations of a 1-h standalone educational intervention.

The audit of eMRs showed they are incomplete repositories for ACP documents. System fragmentation means that valid ACPs may exist but be inaccessible at the point of care because they are uploaded to another health district’s eMR or stored elsewhere, e.g., a patient's home [28], and are not integrated into the medical record [28]. This is particularly pertinent for people residing in aged care facilities, where a resident’s ACP-related documentation is usually completed as it is an Australian care standard [29], but may not be routinely shared with hospital eMR systems, as we have demonstrated [Table 5].

### Limitations

A key limitation was the brief 2–3-month follow-up period, a pragmatic constraint of the study timeframe which may have been insufficient to capture sustained practice change [30]. The pre–post-design without a control group precludes causal inference, and the modest questionnaire sample size, although comparable with other pilot studies [13, 31], limited subgroup power. Ceiling effects from high baselines scores limited the ability to detect true positive effects [32]. Social desirability bias [33] or a Hawthorne effect [34] may have influenced the results, especially the exceptionally large effect size for confidence (*r* = 0.87) despite mitigation measures such as anonymization. The questionnaire was developed for this study and, while it was reviewed by clinicians and a peer worker for face validity, its psychometric properties were not formally assessed.

This study used eMR documentation as the primary outcome and a proxy for measuring change in clinical practice. This may be considered a limitation as ACP discussions may have occurred but not been documented or ACP documents may not have been uploaded to eMR, for reasons discussed above, leading to bias through underreporting. This fragmentation was further highlighted by the lack of integration between the local eMR and the national My Health Record system as no consumer had documents in both systems simultaneously.

### Implications for clinical practice and future research

Integrating qualitative feedback, questionnaire scores, and eMR data revealed the intervention's potential to empower staff to engage consumers in ACP but also reveals specific areas needing attention. Echoing initial qualitative findings [9], participants endorsed practical elements (e.g., clinical vignettes, discussion) and theoretical clarifications (e.g., ACP vs. ACD) of the intervention, likely driving confidence gains. These elements should be retained, but to bridge the gap between improved confidence and clinical action, and as suggested by staff, education should include practical skills training, such as role-play and demonstrations of eMR documentation.

A multifaceted approach to education is recognized as more likely to be effective in changing practice, including strategies, such as targeted peer-led education, small group or individual case-based discussions, practice audit and feedback, and reminder systems [26]. Change champions embedded within healthcare settings may complement educational interventions [35]. Other studies in mental health settings have shown champions motivate behavior change by modeling supportive attitudes [36] and clinical skills/techniques [36], the latter crucial in ACP given the complexity of navigating family concerns and cultural considerations [3], capacity and ongoing symptoms of mental illness [9], and emphasized by participants in this study. Recognizing that organizational change is required to sustain engagement in new clinical practices [27], mental health services should consider embedding ACP into governance processes, such as through eMR audits of documentation and incorporation of ACP into the agenda of clinical team business meetings.

Ensuring the effects of the intervention are sustained is crucial to evaluating cost-effectiveness and overall benefit [27]. Repeating educational interventions can lead to more sustained effects, likely due to repeated exposure [26, 27, 37], and was suggested by our participants. Future studies should move beyond pre–post-designs to employ controlled, longitudinal methodologies with follow-up periods exceeding 12 months to better evaluate sustained behavior change [14, 15]. Learning from a successful approach to improving aged care home resident participation in ACP [38], other strategies to trial in mental health settings may include a train-the-trainer model with more lengthy education seminars involving role-play, and reinforcing learning with quick reference guides, ongoing supervision, and follow-up. The use of digital decision support tools, such as video-based resources [39] tailored for people with mental illness, could enhance understanding of complex ACP concepts. Future work should include consumers and carers in co-designed educational interventions and evaluate the cost-effectiveness of these more intensive implementation strategies [27].

## Conclusion

A brief educational intervention significantly improved the knowledge, attitudes, and confidence of multidisciplinary mental health clinicians and peer workers regarding ACP with older people living with mental illness. The intervention utilized evidence-informed purpose-developed audio–visual resources situated in Australian medicolegal and mental health contexts. Conducted with frontline mental health staff, including peer workers in investigator, educator and participant roles, the intervention was pragmatic, well-received by staff and required minimal resources. The intervention was undertaken in real-world conditions including competing demands for staff time and the logistics of delivering condensed education in hybrid formats within existing programs. Increased preparedness to engage in ACP did not translate into a statistically significant increase in ACP documentation within eMR during the follow-up period. Feedback from participants highlighted a specific need for practical skills training incorporating experiential learning and clearer guidance on documentation processes to operationalize their theoretical knowledge. The resources and educational intervention could readily be adapted for other contexts and legal frameworks and are thus transferable to diverse healthcare settings.

## Supplementary Information

Below is the link to the electronic supplementary material.

Supplementary file1 (DOCX 29 KB)

## Data Availability

The deidentified data supporting the findings of this study may be available from Keanu Crous upon reasonable request if approved by the Concord Hospital Human Research Ethics Committee.

## Footnotes

1 An Advance Care Directive is a legally binding document (under Common Law in NSW) that records a person’s specific preferences for future health care.
