# Supplementary material for: Evaluation of a novel educational intervention for mental health staff on advance care planning with older people with mental illness
Source: Eur Geriatr Med. 2026 Jan 27;17(2):917–27. doi: 10.1007/s41999-026-01416-y (PMC13109282; doi:10.1007/s41999-026-01416-y)
Supplement: Supplementary file 1 — Supplementary file1 (DOCX 29 KB) [file 41999_2026_1416_MOESM1_ESM.docx]

# Supplementary Material

## Box 1: Overview of education session content

| - Definitions and comparison of Advance Care Planning (ACP) and Advance Care Directives (ACD) - Relevance of ACP to mental health clinicians and peer workers - Summary of qualitative research regarding ACP with people with mental illness - Aims of the education session - Turning the identified barriers to ACP into solutions  1. Education and training deficits  - Fears of causing distress - Navigating capacity and supported decision making - Lack of practical skills for ACP (“how to” videos demonstrating starting the conversation and dealing with psychotic symptoms) - Educating consumers and carers (provision of targeted information, cultural and personalised considerations, resources; including “how to” video demonstrating techniques for working with concerned family)  1. Practical considerations  - When, where and how to do ACP - Documentation of ACP in the electronic medical record (eMR)  1. Processes and Systems  - Access to education resources (handouts and online) - Embedding ACP within practice - Who to learn from; champions, role models, supervision, consultation - Leadership - Five Clinical Vignettes - Interactive Discussion of how to manage aspects of ACP, including capacity assessment and family/carer involvement - Summary of key points for practice and links to resources |
| --- |
